# Work Engagement in the Association Between Emotional Intelligence and Intent to Stay Among Registered Nurses in Riyadh: A Cross-Sectional Study

**DOI:** 10.3390/healthcare14203462

**Published:** 2026-10-15

**Authors:** Bandar Majrashi, Mohammad Alharbi, Omar Qaladi

**Affiliations:** 1 College of Nursing, King Saud University, Riyadh 11421, Saudi Arabia; 2 Nursing Administration and Education Department, College of Nursing, King Saud University, Riyadh 11421, Saudi Arabia; moalharbi@ksu.edu.sa; 3 Community and Psychiatric Mental Health Nursing Department, College of Nursing, King Saud University, Riyadh 11421, Saudi Arabia; oqaladi@ksu.edu.sa

**Keywords:** emotional intelligence, work engagement, intent to stay, nurse retention, registered nurses, indirect association, Saudi Arabia

## Abstract

**Background/Objectives:** Research on nurse retention in Saudi Arabia has focused mainly on turnover intention, and little is known about how emotional intelligence and work engagement relate to nurses’ intent to stay. This study examined these associations among registered nurses in Riyadh, including the indirect association involving work engagement. **Methods:** A cross-sectional survey used convenience sampling to recruit 299 registered nurses from two tertiary referral hospitals in Riyadh, Saudi Arabia. Emotional intelligence, work engagement, and intent to stay were measured using the WLEIS, UWES-9, and McCain’s Intent to Stay Scale. Hierarchical regression and bootstrapping indirect-association model (5000 samples) were used, adjusting for sociodemographic and work-related characteristics. **Results:** Emotional intelligence, work engagement, and intent to stay were moderately and positively correlated (*r* = 0.43–0.48). After adjustment, work engagement (β = 0.38) and emotional intelligence (β = 0.24) remained independently associated with intent to stay (both *p* < 0.001), and the final model explained 34.3% of the variance in intent to stay. The total association between emotional intelligence and intent to stay was *B* = 0.365, of which the statistical indirect association involving work engagement was *B* = 0.148 (95% bootstrap CI [0.090, 0.226]). **Conclusions:** Emotional intelligence and work engagement were each associated with greater intent to stay in this convenience sample. Because the data are cross-sectional and drawn from two Riyadh hospitals, these associations do not establish causality or temporal ordering.

## 1. Introduction

Nurses are the largest professional group in the health workforce, and persistent shortages, difficult working conditions, and international mobility continue to challenge the sustainability of health systems worldwide [1]. Retention is particularly important in Saudi Arabia, where healthcare transformation continues alongside a nursing workforce comprising both Saudi and expatriate professionals. A systematic review of nurse turnover in Saudi Arabia identified individual, work-environment, and organizational influences [2], and studies in the Kingdom have linked job satisfaction, organizational commitment, self-efficacy, and the work environment with nurses’ intentions to stay or leave [3,4]. Much of this evidence, however, concerns why nurses intend to leave rather than why they intend to stay, and the two are not simple opposites. In a systematic review of 34 studies, Pressley and Garside [5] identified job satisfaction and organizational commitment as central to nurses’ decisions to remain and argued for greater attention to the factors that encourage nurses to stay, rather than only those that push them to leave. Personal resources that help nurses remain engaged in demanding clinical work are one such set of factors.

One such resource is emotional intelligence, the capacity to perceive, understand, use, and regulate emotions in oneself and others. The Wong and Law model conceptualizes emotional intelligence through self-emotion appraisal, others’ emotion appraisal, use of emotion, and regulation of emotion [6]. These capabilities may be especially relevant in nursing, where clinical work involves emotionally charged interactions, competing demands, and sustained exposure to patient and family distress. In Saudi Arabia, emotional intelligence has been positively associated with work engagement among nurses [7], and recent research has linked it with lower burnout and greater engagement [8]. Emotional intelligence has also been positively related to intent to stay among nurses in Jordan [9].

The Job Demands–Resources (JD-R) framework provides the rationale for examining work engagement alongside emotional intelligence. The framework describes a motivational process in which resources foster work engagement, a positive, fulfilling work-related state characterized by vigor, dedication, and absorption [10], and engagement is in turn associated with favorable organizational outcomes such as commitment and lower turnover intention [11,12]. Xanthopoulou et al. [13] extended the model to personal resources, defined as individuals’ sense of their ability to control and influence their environment, and showed that these were associated with engagement. Emotional intelligence fits this definition: the ability to recognize, understand, and regulate emotions helps nurses manage emotionally demanding clinical interactions and has been positively associated with engagement in nursing samples [7,14]. Work engagement, in turn, is consistently associated with retention-related attitudes; a meta-analysis of 40 studies involving 23,451 nurses found a moderate inverse association between engagement and turnover intention [15], and engagement has been examined alongside resilience as a correlate of nurses’ turnover intentions [16]. The JD-R framework therefore positions emotional intelligence as a personal resource and work engagement as a state that lies conceptually between personal resources and retention-related attitudes. In the present cross-sectional study, this ordering is theoretical and is not tested; the indirect analysis estimates a statistical association that is consistent with, but cannot confirm, the proposed sequence.

Previous studies have examined parts of this model. In Saudi Arabia, emotional intelligence has been associated with work engagement [7], and with lower turnover intention, including through its association with job stress [17,18]. Outside Saudi Arabia, Catrin et al. [19] examined emotional intelligence, work engagement, and turnover intention together in a single hospital. The present study does not introduce new constructs; its contribution is more specific. First, it examines intent to stay rather than turnover intention, an outcome that reflects nurses’ positive commitment to remain and is not simply the inverse of intention to leave [5]. Second, it examines these relationships in Saudi tertiary hospitals, where the nursing workforce combines Saudi and expatriate nurses and where evidence on emotional intelligence and positive retention outcomes is limited. Third, it estimates the direct and indirect associations within a single model adjusted for sociodemographic and work-related characteristics. Accordingly, this study examined the associations among emotional intelligence, work engagement, and intent to stay among registered nurses in two tertiary referral hospitals in Riyadh, Saudi Arabia, and evaluated whether the association between emotional intelligence and intent to stay included a statistically significant indirect component involving work engagement.

Four hypotheses were tested: emotional intelligence would be positively associated with work engagement (H1) and with intent to stay (H2); work engagement would be positively associated with intent to stay (H3); and the association between emotional intelligence and intent to stay would include a statistically significant indirect component involving work engagement (H4). Consistent with the cross-sectional design, these hypotheses concern associations, not causal effects or temporal ordering.

## 2. Materials and Methods

### 2.1. Study Design

This study used a quantitative, cross-sectional, correlational design to examine the relationships among emotional intelligence, work engagement, and intent to stay among registered nurses. A cross-sectional design was appropriate for estimating associations among emotional intelligence, work engagement, and intent to stay a single time point, but it cannot establish temporal ordering or mediation. The indirect association estimated in this study is therefore reported as a statistical decomposition of the association between emotional intelligence and intent to stay, not as evidence of a causal mechanism. The study is reported in accordance with the Strengthening Reporting of Observational Studies in Epidemiology (STROBE) guidelines.

### 2.2. Setting

The study was conducted at two government tertiary referral hospitals in Riyadh, Saudi Arabia: King Saud University Medical City (KSUMC) is a university-affiliated academic medical center with a capacity of approximately 1800 beds across its hospitals, and King Saud Medical City (KSMC) is a Ministry of Health tertiary referral hospital with approximately 1400 beds. Including hospitals under two governance arrangements (academic and Ministry of Health) broadened the range of organizational contexts represented in the sample. Participants were recruited from inpatient wards, critical care and emergency units, pediatric and maternity units, dialysis units, and ambulatory care services. In accordance with the conditions of ethical approval, organizational and employment conditions were not compared directly in the present study.

### 2.3. Sample and Sampling

Registered nurses were recruited using convenience sampling. The minimum required sample size was calculated using G*Power 3.1 for multiple linear regression [20]. Assuming an effect size (f^2^ = 0.05), α = 0.05, 80% power, two focal independent variables, and 11 independent variables in the full model, the minimum required sample was 196 participants. This value was chosen as a conservative estimate between Cohen’s conventions for small (0.02) and medium (0.15) effects, given the consistent positive associations reported between emotional intelligence, work engagement, and retention-related outcomes [7,9,15]. To allow for approximately 20% incomplete or unusable responses, the recruitment target was increased to at least 235 nurses. The final analytic sample comprised 299 participants, which exceeded both the minimum required sample and the planned recruitment target. The 11 independent variables specified for the full model were years of nursing experience, gender, marital status, nationality, educational level, current position, three dummy-coded work-unit variables, emotional intelligence, and work engagement. Emotional intelligence and work engagement were the two focal independent variables for the incremental R^2^ calculation. The final sample of 299 provided 80% power to detect an increment as small as f^2^ = 0.033, although a larger sample does not offset the selection bias of convenience sampling.

### 2.4. Participants

The target population was registered nurses working at the two participating hospitals. Nurses were eligible if they were currently employed as registered nurses during the data collection period, had at least one year of nursing experience, and agreed to participate voluntarily. Nurses who did not meet these criteria or declined consent were excluded. All participants were full-time employees, as the hospitals do not employ part-time, locum, or agency nurses; contract type was not recorded. No participants were excluded after recruitment, and there were no missing data.

### 2.5. Data Collection Procedure

Data were collected from April to August 2026 using an electronic, self-administered questionnaire in English, created with Google Forms. The questionnaire was administered in English, the official language of clinical practice and documentation in Saudi hospitals. The original English versions of all three instruments were used. English is the language of nursing education, licensure, and clinical documentation in Saudi Arabia and the common working language of this largely expatriate workforce. The survey was distributed through nurses’ institutional work emails, which included information about the study and the eligibility criteria, and through recruitment posters displayed in the participating clinical departments. The posters contained participant information and a QR code linking to the electronic survey. Because recruitment involved both email distribution and open QR-code access within the participating departments, the exact number of nurses who received, viewed, or accessed the invitation could not be determined; therefore, a conventional response rate could not be calculated. The resulting risk of non-response and self-selection bias is acknowledged in limitation section. A total of 299 eligible complete responses were included in the final analysis. The first page explained the purpose of the study, the voluntary nature of participation, confidentiality, and the estimated completion time of 10–15 min. Participants provided electronic consent before starting the questionnaire, and no personal identifiers were collected. Before data collection, the questionnaire was pilot-tested with 30 registered nurses to assess clarity and completion time; no changes were required, and pilot responses were not included in the analysis. No formal cultural adaptation was undertaken.

### 2.6. Instruments

The questionnaire consisted of four sections: sociodemographic and work-related characteristics, emotional intelligence, work engagement, and intent to stay. This first section collected data on age, gender, marital status, nationality, educational level, years of nursing experience, current position, and work unit.

#### 2.6.1. Wong and Law Emotional Intelligence Scale

Emotional intelligence was measured using the Wong and Law Emotional Intelligence Scale (WLEIS), developed by Wong and Law [6]. The scale consists of 16 items across four dimensions: self-emotion appraisal, others’ emotion appraisal, use of emotion, and regulation of emotion. Each item is rated on a 7-point Likert scale ranging from 1 (strongly disagree) to 7 (strongly agree). Higher mean scores indicate higher emotional intelligence. The WLEIS has shown good reliability and validity in nursing populations [7,21]. In the present study, Cronbach’s alpha was 0.96 (subscales 0.90–0.92), which may partly reflect item redundancy (mean inter-item *r* = 0.61). Sampling adequacy was excellent (Kaiser–Meyer–Olkin [KMO] = 0.95), item–total correlations ranged from 0.69 to 0.83, and one dominant component explained 63.3% of the variance, supporting use of the total score.

#### 2.6.2. Utrecht Work Engagement Scale

Work engagement was measured using the nine-item Utrecht Work Engagement Scale (UWES-9) developed by Schaufeli et al. [10]. The scale comprises three dimensions: vigor, dedication, and absorption, with three items each. Items are rated on a 7-point scale ranging from 0 (never) to 6 (always), and higher mean scores indicate greater engagement. The UWES-9 has been widely used among nurses and has demonstrated acceptable psychometric properties [14,22]. In the present study, the UWES-9 demonstrated acceptable overall internal consistency (Cronbach’s α = 0.88). However, the absorption subscale showed lower reliability (α = 0.60); therefore, findings related to this subscale should be interpreted cautiously. The primary analyses were based on the total UWES-9 score.

#### 2.6.3. McCain Intent to Stay Scale

Intent to stay was measured using McCain’s Intent to Stay Scale, a five-item measure that has been used previously among nurses in Saudi Arabia [23,24,25]. The scale assesses nurses’ perceived likelihood of remaining in their current job. Each item is rated on a five-point Likert scale ranging from 1 (strongly disagree) to 5 (strongly agree). Item scores are summed and divided by the number of items to obtain a mean score, with higher scores indicating a stronger intent to stay. In the present study, Cronbach’s α was 0.92 (KMO = 0.88; item–total correlations 0.70–0.84; one component explaining 75.7% of the variance). The scale measures intended rather than actual retention.

### 2.7. Data Analysis

Data were analyzed using SPSS (v. 30; IBM Corp., Armonk, NY, USA), and the PROCESS Model 4 was used to estimate the statistical indirect association between emotional intelligence and intent to stay via work engagement. Emotional intelligence was specified as the independent variable (X), work engagement as the specified (M), and intent to stay as the dependent variable (Y). The indirect effect was estimated using 5000 bootstrap samples and considered statistically supported when the 95% bootstrap confidence interval did not include zero. Because the data were cross-sectional, the indirect effect was interpreted as a statistical association rather than evidence of temporal or causal mediation. The covariates entered in the adjusted model were years of nursing experience, gender, marital status, nationality, educational level, current position, and work unit; categorical variables were entered using indicator coding with the reference categories. These variables were selected to account for key demographic and occupational characteristics potentially associated with intent to stay and the focal study variables, without implying that they represent established causal confounders. Before analysis, the dataset was screened for duplicate submissions, missing values, outliers, and distributional assumptions. No missing data were identified. Descriptive statistics were used to summarize participant characteristics and study variables, and internal consistency was evaluated using Cronbach’s alpha. Structural evidence for each instrument in the present sample was examined using the KMO measure, principal component analysis, and corrected item–total correlations. Pearson’s correlation coefficient was used to assess associations among continuous variables, including age, years of experience, emotional intelligence, work engagement, and intent to stay. Welch’s *t*-test was used to compare study variables between two groups (e.g., gender and nationality), and one-way analysis of variance (ANOVA) with Tukey’s post hoc test was used for variables with more than two groups (e.g., position and work unit). Multiple linear regression was conducted to identify factors associated with intent to stay. Variables were entered hierarchically in two blocks: the sociodemographic and work-related covariates in Block 1, and emotional intelligence and work engagement in Block 2. The increment in explained variance was evaluated using the change in R^2^ and the associated F-change test, and multicollinearity was assessed using variance inflation factors. Significance was set at *p* < 0.05. Years of nursing experience was entered instead of age because the two were strongly correlated (r = 0.75). Regression assumptions were checked using the RESET test (linearity), the Breusch–Pagan test (homoscedasticity), residual skewness and kurtosis, Cook’s distance, and VIFs. Because emotional intelligence was skewed, the main models were re-estimated with HC3 robust standard errors and after excluding extreme emotional intelligence scores (|z| > 3.29), and Spearman correlations were also calculated. Holm-adjusted *p*-values were reported for the 18 group comparisons. Significance was set at *p* < 0.05.

## 3. Results

### 3.1. Participant Characteristics

A total of 299 registered nurses were included in the analysis. Their mean age was 34.07 years (SD ± 5.97; range 23–56), and they had an average of 9.79 years of nursing experience (SD ± 4.52). Most were women (*n* = 250, 83.6%), and just over half were married (*n* = 155, 51.8%). Non-Saudi nurses made up 53.5% of the sample (*n* = 160). Nearly all participants held a bachelor’s degree (*n* = 284, 95.0%), and only 15 (5.0%) had a postgraduate qualification. Staff nurses were the largest group (*n* = 210, 70.2%), followed by charge nurses (*n* = 48, 16.1%) and nurses in management or other roles (*n* = 41, 13.7%). Participants came mainly from critical care and emergency units (*n* = 108, 36.1%) and inpatient wards (*n* = 106, 35.5%), with smaller numbers from pediatrics and maternity (*n* = 48, 16.1%) and dialysis and ambulatory care (*n* = 37, 12.4%) as shown in Table 1.

### 3.2. Descriptive Statistics and Reliability

Nurses scored above the midpoint on all three measures (Table 1). The mean score was 5.81 out of 7 for emotional intelligence, 4.33 out of 6 for work engagement, and 3.85 out of 5 for intent to stay. Emotional intelligence scores were concentrated near the top of the scale (skewness = −2.19; kurtosis = 7.69). This restricted variability at the upper end of the emotional intelligence scale may have reduced discrimination among participants with high scores and may have influenced the magnitude of associations involving emotional intelligence; these findings should therefore be interpreted cautiously. All three scales showed good-to-excellent reliability (α = 0.88–0.96). The particularly high internal consistency of the emotional intelligence scale should therefore be interpreted as strong reliability while recognizing the possibility of high item homogeneity.

### 3.3. Bivariate Associations

Emotional intelligence, work engagement, and intent to stay were positively and moderately intercorrelated (Table 2). Emotional intelligence was associated with both work engagement (r = 0.48) and intent to stay (r = 0.43), and work engagement was associated with intent to stay (r = 0.48; all *p* < 0.001). Neither age nor years of experience was significantly correlated with any of the study variables. These correlations represent cross-sectional associations and do not indicate directionality.

Several differences were observed across participant characteristics (Table 3). Compared with Saudi nurses, non-Saudi nurses reported significantly higher emotional intelligence (*p* = 0.010) and intent to stay (*p* < 0.001). Married nurses also reported greater intent to stay than their unmarried counterparts (*p* = 0.041). Nurses holding postgraduate qualifications reported lower work engagement (*p* = 0.013) and intent to stay (*p* = 0.047); however, these estimates should be interpreted cautiously given the small size of this subgroup (*n* = 15). Work engagement varied significantly by clinical unit (F = 4.97, *p* = 0.002). After Holm adjustment for 18 comparisons, only the differences in intent to stay by nationality (adjusted *p* = 0.009) and in work engagement by clinical unit (adjusted *p* = 0.038) remained significant; the others, including those involving the 15 postgraduate nurses, should be regarded as exploratory. No significant differences were found by gender or current position.

### 3.4. Multivariable Regression Analysis

Variables were entered hierarchically in two blocks. The sociodemographic and work-related covariates entered in Block 1 explained 9.5% of the variance in intent to stay (*R*^2^ = 0.095, adjusted *R*^2^ = 0.066, *F*(9, 289) = 3.35, *p* < 0.001). Adding emotional intelligence and work engagement in Block 2 significantly increased the explained variance (Δ*R*^2^ = 0.249, *F*-change(2, 287) = 54.40, *p* < 0.001), and the final model was statistically significant, *F*(11, 287) = 13.65, *p* < 0.001, explaining 34.3% of the variance in intent to stay (*R*^2^ = 0.343, adjusted *R*^2^ = 0.318), so most of the variance is attributable to factors not measured in this study. After adjustment for sociodemographic and work-related characteristics, both work engagement (β = 0.38, *p* < 0.001) and emotional intelligence (β = 0.24, *p* < 0.001) remained significant independent factors associated with intent to stay (Table 4). Nurses working in critical care and emergency units reported lower intent to stay than those on inpatient wards (*p* = 0.019). Postgraduate education was also associated with lower intent to stay (*p* = 0.020); because this estimate rests on a small subgroup (*n* = 15), it should be considered exploratory and interpreted with caution. Nationality was not significant after adjustment. Diagnostics showed no evidence of non-linearity (RESET *p* = 0.82), heteroscedasticity (Breusch–Pagan *p* = 0.15), or influential observations (maximum Cook’s distance = 0.06). Results for emotional intelligence and work engagement were unchanged with HC3 robust standard errors, although postgraduate education was no longer significant (*p* = 0.11), and Spearman correlations were similar to Pearson correlations (ρ = 0.44–0.54).

### 3.5. Direct and Indirect Association Analysis

**Figure 1:**
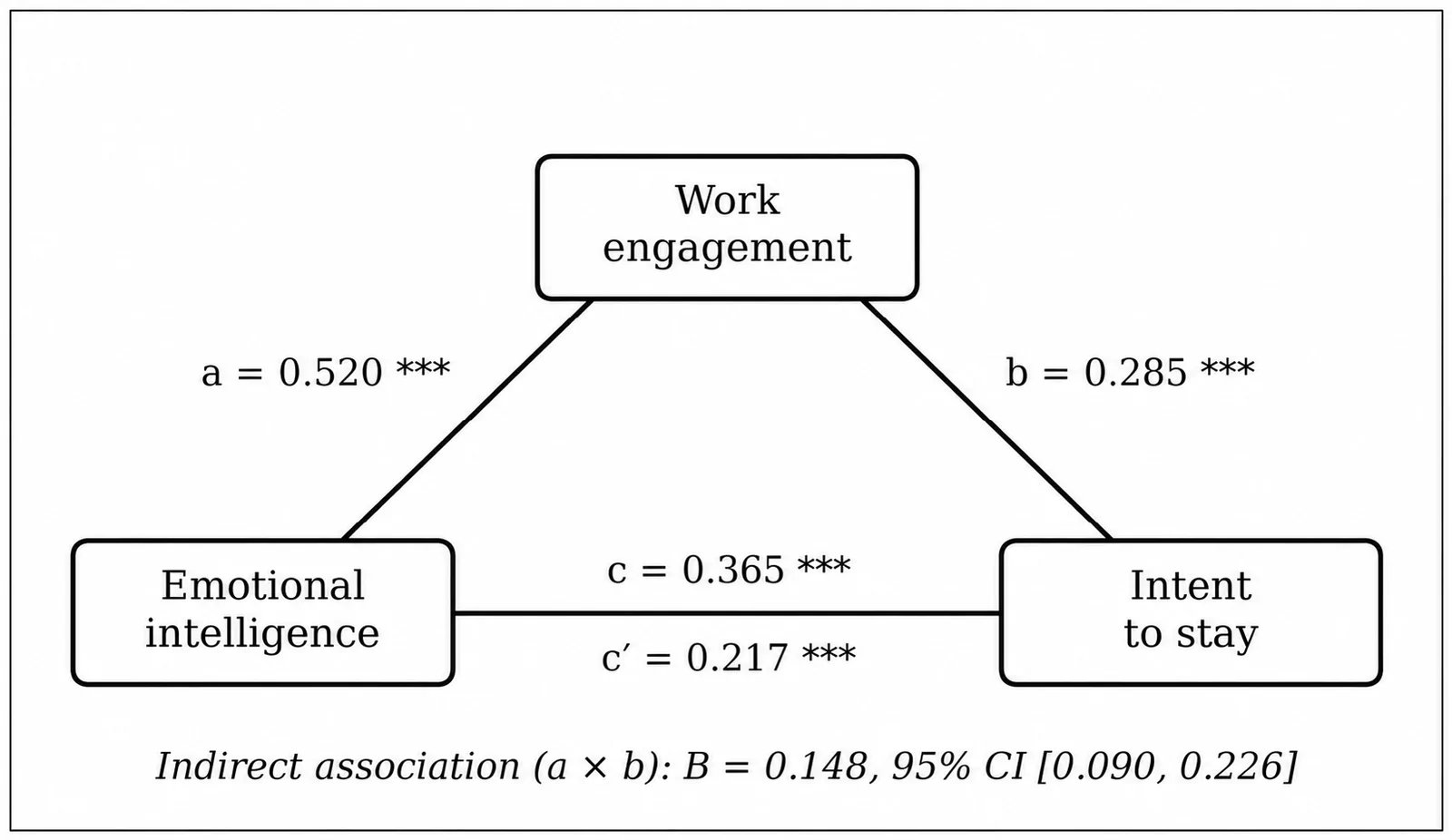
Indirect association between emotional intelligence and intent to stay involving work engagement (*N* = 299), *** *p* < 0.001.

A significant statistical indirect association between emotional intelligence and intent to stay involving work engagement was observed (Table 5). After adjustment for sociodemographic and work-related characteristics, emotional intelligence was positively associated with work engagement (B = 0.520, SE = 0.062, 95% CI [0.398, 0.642], *p* < 0.001), and work engagement was positively associated with intent to stay after accounting for emotional intelligence (B = 0.285, SE = 0.043, 95% CI [0.200, 0.370], *p* < 0.001). The bootstrapped indirect association was B = 0.148 (BootSE = 0.035, 95% bootstrap CI [0.090, 0.226]). Emotional intelligence also remained directly associated with intent to stay after work engagement was included in the model (B = 0.217, SE = 0.051, 95% CI [0.117, 0.316], *p* < 0.001). These findings are consistent with a statistically significant indirect association; however, because the data were collected cross-sectionally, the indirect association may reflect shared covariance among the three variables and does not establish temporal ordering or causal mediation. The model coefficients are presented in Figure 1.

## 4. Discussion

This study examined emotional intelligence, work engagement, and intent to stay among registered nurses in two tertiary hospitals in Riyadh. Both emotional intelligence and work engagement were independently associated with intent to stay, and part of the association between emotional intelligence and intent to stay was statistically accounted for by work engagement. Because the design was cross-sectional, these findings describe associations rather than a process.

The positive relationship between emotional intelligence and work engagement is consistent with findings across several nursing populations. A study conducted by Zhu et al. [26] reported that emotional intelligence was positively related to work engagement among Chinese registered nurses and further showed that organizational justice contributed to this relationship. Pérez-Fuentes et al. [21] similarly found significant relationships between emotional-intelligence dimensions and vigor, dedication, and absorption among Spanish nurses. In Saudi Arabia, Turjuman and Alilyyani [7] found a positive relationship between emotional intelligence and work engagement among Saudi nurses, while Aqtam et al. [22] identified a similar association among Palestinian neonatal intensive care nurses. Collectively, these studies suggest that nurses who perceive themselves as better able to recognize, use, and regulate emotions also tend to report greater energy and psychological involvement in their work.

Emotional intelligence was also positively associated with intent to stay. This finding corresponds with Al-Hamdan et al. [9], who demonstrated a positive relationship between emotional intelligence and intent to stay among nurses employed in Jordanian hospitals, providing particularly relevant evidence from another Middle Eastern healthcare system. Studies examining the related construct of turnover intention generally support the same direction of association. For example, Altalhi and Alilyyani [18] found that higher emotional intelligence was associated with lower intention to leave among emergency nurses in Saudi Arabia. More recently, Alilyyani [17] reported a significant indirect association involving job stress between emotional intelligence and turnover intention among Saudi nurses. Taken together, these findings suggest that the association between emotional intelligence and retention-related attitudes may coexist with several psychological and occupational factors rather than reflect a single explanatory process. At the same time, emotional intelligence should not be considered independently from the organizational environment. In a systematic review of nurse turnover in Saudi Arabia, Albalawi et al. [2] identified individual, work-environment, and organizational factors as key influences on nurses’ decisions to remain in or leave their employment.

In the present study, work engagement showed the strongest standardized association with intent to stay in the regression model, consistent with a growing body of research identifying engagement as an important correlate of nurse retention. In meta-analysis study conducted by Zhu et al. [15] synthesized 40 studies involving 23,451 nurses and found a moderate inverse association between work engagement and turnover intention, indicating that more engaged nurses were generally less likely to consider leaving. Notably, this association varied by cultural background and economic context, which highlights the value of examining these relationships in settings such as Saudi Arabia. Similarly, in a study of 462 nurses in Ghana, Poku et al. [16] found that work engagement was negatively associated with turnover intention and reported a significant indirect association involving resilience. Furthermore, a systematic review of 34 studies by Pressley and Garside [5] concluded that nurses’ intention to stay is shaped by individual, relational, and environmental factors, particularly job satisfaction and organizational commitment. However, the larger coefficient for engagement should not be taken to mean that it is inherently more important than emotional intelligence. Engagement reflects nurses’ current experience of their work and is therefore conceptually closer to decisions about staying, whereas emotional intelligence may represent a more distal personal resource whose association with retention attitudes may coexist with engagement, resilience, and coping with workplace stress.

The study also found a significant indirect statistical association between emotional intelligence and intent to stay involving work engagement (B = 0.148, 95% bootstrap CI [0.090, 0.226]), although its interpretation is limited by the cross-sectional design. This finding is consistent with Catrin et al. [19], who reported a significant indirect association involving work engagement between emotional intelligence and turnover intention, and extends their work by focusing on intent to stay among nurses in Saudi Arabia. Within the Job Demands–Resources framework, emotional intelligence may be conceptualized as a personal resource that is positively associated with work engagement [13]. However, work engagement is unlikely to be the only variable involved in the observed association between emotional intelligence and retention-related attitudes. Among Saudi nurses, Alilyyani [17] reported a significant indirect association involving job stress between emotional intelligence and turnover intention, while Alshammari et al. [8] reported an indirect association involving burnout between emotional intelligence and work engagement. Emotional intelligence has also been examined in relation to organizational commitment and turnover intention [27]. The remaining direct association in the present model suggests that other unmeasured psychological or organizational factors may also contribute to the observed relationship. These adjusted associations should be interpreted in light of potential residual confounding from unmeasured organizational and occupational factors.

Two additional findings warrant cautious interpretation. Nurses in critical care and emergency services reported lower adjusted intent to stay, which is consistent with meta-analytic evidence of high turnover intention in intensive care [28] and emergency nurses [29]; the working conditions that might explain this difference were not measured. Nurses with postgraduate qualifications also reported lower intent to stay, but this estimate was based on only 15 participants and was not significant with robust standard errors, so it should be regarded as exploratory.

### 4.1. Study Limitations

Several limitations should be considered. First, the cross-sectional design prevents determination of temporal sequence or causal relationships. Although the analysis identified a statistically significant indirect association involving work engagement, it cannot demonstrate that emotional intelligence precedes engagement or that engagement subsequently increases intent to stay. Second, residual confounding also cannot be excluded because several theoretically relevant organizational and occupational factors, including workload, staffing adequacy, leadership, organizational support, burnout, job satisfaction, and career-development opportunities, were not measured in the present study. Third, convenience sampling from two tertiary referral hospitals limits representativeness and restricts generalization to nurses working in other hospitals or regions of Saudi Arabia. A response rate could not be calculated, so non-response and self-selection bias cannot be excluded, and hospital-level clustering could not be examined because hospital was not recorded. Fourth, all principal variables were assessed via self-report within the same questionnaire, introducing potential common-method and social-desirability bias. The English-only questionnaire was pilot-tested but not culturally adapted. Fifth, the skewed distribution of emotional intelligence restricted its variance and may have attenuated its associations, and the absorption subscale had low reliability (α = 0.60). Finally, intent to stay is an attitudinal outcome rather than observed employment retention, and the extent to which it corresponds to actual turnover in this setting is unknown.

### 4.2. Study Implications and Future Directions

The findings suggest that emotional intelligence and work engagement may be relevant considerations for nursing management, although intervention effects cannot be inferred from the present design. Because higher emotional intelligence and work engagement were associated with stronger intent to stay, both may be useful to monitor alongside established retention indicators; whether strengthening them would improve retention has not been tested. However, retention should not be framed primarily as an individual nurse responsibility. Albalawi et al. [2] showed that nurse turnover in Saudi Arabia is influenced by individual, work-environment, and organizational factors, and Pressley and Garside [5] similarly highlighted the combined influence of individual, relational, and environmental factors on nurses’ decisions to remain. Any initiatives targeting emotional skills or engagement would require evaluation and should complement, rather than replace, strategies addressing staffing, workload, recognition, supportive leadership, career development, and workplace conditions. Future research should prioritize longitudinal and multicenter designs, include organizational determinants such as workload, staffing, leadership, and job satisfaction, and examine actual retention outcomes in addition to intent to stay.

## 5. Conclusions

In a convenience sample of nurses from two tertiary hospitals in Riyadh, emotional intelligence and work engagement were each independently associated with stronger intent to stay, and part of the association between emotional intelligence and intent to stay was statistically accounted for by work engagement. These cross-sectional associations do not establish causality or temporal order, and they may not generalize beyond similar hospital settings.

## Figures and Tables

**Table 1:** Participant characteristics and descriptive statistics of study variables (*N* = 299).

Characteristic/Variable	*n* (%) or Mean ± SD	Range	α
**Participant characteristics**			
Age, years	34.07 ± 5.97	23–56	
Gender			
Female	250 (83.6)		
Male	49 (16.4)		
Marital status			
Married	155 (51.8)		
Unmarried	144 (48.2)		
Nationality			
Saudi	139 (46.5)		
Non-Saudi	160 (53.5)		
Years of nursing experience	9.79 ± 4.52	1–26	
Education level			
Diploma or bachelor’s degree	284 (95.0)		
Postgraduate (master’s/doctoral)	15 (5.0)		
Current position			
Staff nurse	210 (70.2)		
Charge nurse	48 (16.1)		
Nursing management/other	41 (13.7)		
Work unit			
Inpatient wards	106 (35.5)		
Critical care and emergency	108 (36.1)		
Pediatrics and maternity	48 (16.1)		
Dialysis and ambulatory care	37 (12.4)		
***Study measures***			
Emotional intelligence (16 items)	5.81 ± 0.89	1–7	0.96
Self-emotion appraisal (4 items)	5.85 ± 0.98	1–7	0.90
Others’ emotion appraisal (4 items)	5.73 ± 1.02	1–7	0.90
Use of emotion (4 items)	5.88 ± 0.97	1–7	0.91
Regulation of emotion (4 items)	5.78 ± 1.03	1–7	0.92
Work engagement (9 items)	4.33 ± 1.08	0–6	0.88
Vigor (3 items)	4.05 ± 1.23	0–6	0.71
Dedication (3 items)	4.66 ± 1.26	0–6	0.86
Absorption (3 items)	4.27 ± 1.15	0–6	0.60
Intent to stay (5 items)	3.85 ± 0.82	1–5	0.92

Note. Cronbach’s α values are reported for total scales and individual subscales; subscale reliability should be interpreted separately from total-scale reliability.

**Table 2:** Pearson correlations among continuous study variables (*N* = 299).

Variable	1	2	3	4	5
1. Age	—				
2. Years of experience	0.75 ***	—			
3. Emotional intelligence	0.04	0.01	—		
4. Work engagement	−0.04	−0.06	0.48 ***	—	
5. Intent to stay	0.06	0.10	0.43 ***	0.48 ***	—

Pearson correlation coefficients based on total scale scores. *** *p* < 0.001 (two-tailed).

**Table 3:** Differences in emotional intelligence, work engagement, and intent to stay by participant characteristics (*N* = 299).

Characteristic	*n*	Emotional Intelligence	Work Engagement	Intent to Stay
Gender				
Female	250	5.85 ± 0.87	4.33 ± 1.11	3.85 ± 0.80
Male	49	5.65 ± 0.98	4.31 ± 0.90	3.81 ± 0.89
Welch’s *t* test		*t* = 1.33, *p* = 0.190	*t* = 0.13, *p* = 0.897	*t* = 0.33, *p* = 0.742
Marital status				
Married	155	5.84 ± 0.99	4.29 ± 1.07	3.94 ± 0.79
Unmarried	144	5.78 ± 0.79	4.37 ± 1.09	3.75 ± 0.83
Welch’s *t* test		*t* = 0.53, *p* = 0.594	*t* = −0.66, *p* = 0.507	*t* = 2.05, *p* = 0.041
Nationality				
Saudi	139	5.67 ± 1.10	4.20 ± 1.25	3.67 ± 0.90
Non-Saudi	160	5.94 ± 0.64	4.44 ± 0.89	4.00 ± 0.70
Welch’s *t* test		*t* = −2.59, *p* = 0.010	*t* = −1.94, *p* = 0.053	*t* = −3.53, *p* < 0.001
Education level				
Diploma or bachelor’s	284	5.84 ± 0.87	4.37 ± 1.06	3.88 ± 0.77
Postgraduate	15	5.31 ± 1.26	3.53 ± 1.14	3.15 ± 1.30
Welch’s *t* test		*t* = 1.62, *p* = 0.127	*t* = 2.79, *p* = 0.013	*t* = 2.17, *p* = 0.047
Current position				
Staff nurse	210	5.77 ± 0.84	4.24 ± 1.11	3.82 ± 0.79
Charge nurse	48	5.98 ± 1.05	4.54 ± 0.99	3.95 ± 0.86
Nursing management/other	41	5.86 ± 0.99	4.53 ± 0.97	3.83 ± 0.90
One-way ANOVA		*F* = 1.13, *p* = 0.325	*F* = 2.29, *p* = 0.103	*F* = 0.46, *p* = 0.631
Work unit				
Inpatient wards	106	5.68 ± 1.10	4.18 ± 1.15	3.85 ± 0.77
Critical care and emergency	108	5.92 ± 0.68	4.55 ± 0.90	3.80 ± 0.83
Pediatrics and maternity	48	5.90 ± 0.58	4.51 ± 0.92	3.91 ± 0.69
Dialysis and ambulatory care	37	5.76 ± 1.07	3.87 ± 1.34	3.89 ± 1.06
One-way ANOVA		*F* = 1.47, *p* = 0.224	*F* = 4.97, *p* = 0.002	*F* = 0.27, *p* = 0.847

Note. Subgroup comparisons were exploratory; findings for the postgraduate group should be interpreted cautiously because of the small sample (*n* = 15).

**Table 4:** Multiple linear regression of factors associated with intent to stay (*N* = 299).

Variable	*B*	SE	95% CI	*β*	*t*	*p*
(Constant)	1.331	0.306	0.73 to 1.93	—	4.36	<0.001
Years of nursing experience	0.014	0.010	−0.01 to 0.03	0.08	1.34	0.181
Male (ref. female)	0.158	0.115	−0.07 to 0.38	0.07	1.38	0.169
Married (ref. unmarried)	0.106	0.096	−0.08 to 0.30	0.06	1.11	0.270
Saudi (ref. non-Saudi)	−0.149	0.095	−0.34 to 0.04	−0.09	−1.56	0.120
Postgraduate (ref. diploma/bachelor’s)	−0.447	0.191	−0.82 to −0.07	−0.12	−2.34	0.020
Supervisory role (ref. staff nurse)	−0.050	0.098	−0.24 to 0.14	−0.03	−0.51	0.607
Work unit (ref. inpatient wards)						
Critical care and emergency	−0.224	0.095	−0.41 to −0.04	−0.13	−2.37	0.019
Pediatrics and maternity	−0.063	0.122	−0.30 to 0.18	−0.03	−0.52	0.607
Dialysis and ambulatory care	0.034	0.143	−0.25 to 0.31	0.01	0.24	0.811
Emotional intelligence	0.217	0.051	0.12 to 0.32	0.24	4.29	<0.001
Work engagement	0.285	0.043	0.20 to 0.37	0.38	6.62	<0.001

Note. *B* = unstandardized regression coefficient; SE = standard error; *β* = standardized regression coefficient; CI = confidence interval. The overall regression model was statistically significant, R^2^ = 0.343, adjusted R^2^ = 0.318, F(11, 287) = 13.65, *p* < 0.001. Variance inflation factors were ≤1.51, indicating no evidence of problematic multicollinearity. Age was not entered simultaneously with years of nursing experience because the two variables were strongly correlated (*r* = 0.75).

**Table 5:** Total, direct, and statistical indirect associations between emotional intelligence and intent to stay (*N* = 299).

Effect	Path	*B*	SE	95% CI	*t*	*p*
EI with work engagement	*a*	0.520	0.062	0.398 to 0.642	8.40	<0.001
Work engagement with intent to stay (adjusted for EI)	*b*	0.285	0.043	0.200 to 0.370	6.62	<0.001
EI with intent to stay: total association	*c*	0.365	0.049	0.270 to 0.460	7.52	<0.001
EI with intent to stay: direct association	*c′*	0.217	0.051	0.117 to 0.316	4.29	<0.001
Statistical indirect association involving work engagement	*a × b*	0.148	0.035	0.090 to 0.226	—	—

Note. EI, emotional intelligence; *B*, unstandardized coefficient; SE, standard error; CI, confidence interval. All paths adjusted for sociodemographic and work variables. Indirect effect CI based on 5000 bootstrap samples. Because all variables were measured cross-sectionally, the indirect effect is interpreted as a statistical indirect association and not as evidence of temporal or causal mediation.

## Data Availability

The data presented in this study are available from the corresponding author upon reasonable request. The data are not publicly available due to privacy and ethical restrictions related to the protection of research participants.

## Abbreviations

EI	Emotional Intelligence
KSMC	King Saud Medical City
KSUMC	King Saud University Medical City
STROBE	Strengthening the Reporting of Observational Studies in Epidemiology
UWES-9	Utrecht Work Engagement Scale-9
WLEIS	Wong and Law Emotional Intelligence Scale

## References

[1] WHO (2025). State of the World’s Nursing 2025: Investing in Education, Jobs, Leadership and Service Delivery.

[2] Albalawi A.M., Pascua G.P., Alsaleh S.A., Sabry W.M., Ahajan S.N., Abdulla J., Abdulalim A., Salih S.S. (2024). Factors Influencing Nurses Turnover in Saudi Arabia: A Systematic Review. Nurs. Forum.

[3] Alshaibani N.M., Aboshaiqah A.E., Alanazi N.H. (2024). Association of Job Satisfaction, Intention to Stay, Organizational Commitment, and General Self-Efficacy Among Clinical Nurses in Riyadh, Saudi Arabia. Behav. Sci..

[4] Alharbi A.A., Alharbi A.A., Dahinten V.S., MacPhee M. (2020). The Relationships between Nurses’ Work Environments and Emotional Exhaustion, Job Satisfaction, and Intent to Leave among Nurses in Saudi Arabia. J. Adv. Nurs..

[5] Pressley C., Garside J. (2023). Safeguarding the Retention of Nurses: A Systematic Review on Determinants of Nurse’s Intentions to Stay. Nurs. Open.

[6] Wong C.-S., Law K.S. (2002). The Effects of Leader and Follower Emotional Intelligence on Performance and Attitude. Leadersh. Q..

[7] Turjuman F., Alilyyani B. (2023). Emotional Intelligence among Nurses and Its Relationship with Their Performance and Work Engagement: A Cross-Sectional Study. J. Nurs. Manag..

[8] Alshammari B., Pangket P., Alrasheeday A.M., Baghdadi N.A., Alkubati S., Cabansag D.I., Gugoy N., Alshammari S.M., Alanazi A., Alanezi M. (2025). The Mediating Role of Burnout in the Relationship Between Emotional Intelligence and Work Engagement Among Hospital Nurses: A Structural Equation Modeling Approach. Nurs. Rep..

[9] Al-Hamdan Z., Muhsen A., Alhamdan M., Rayan A., Banyhamdan K., Bawadi H. (2019). Emotional Intelligence and Intent to Stay among Nurses Employed in Jordanian Hospitals. J. Nurs. Manag..

[10] Schaufeli W.B., Bakker A.B., Salanova M. (2006). The Measurement of Work Engagement with a Short Questionnaire: A Cross-National Study. Educ. Psychol. Meas..

[11] Bakker A.B., Demerouti E., Sanz-Vergel A. (2023). Job Demands–Resources Theory: Ten Years Later. Annu. Rev. Organ. Psychol. Organ. Behav..

[12] Bakker A.B., Demerouti E. (2017). Job Demands–Resources Theory: Taking Stock and Looking Forward. J. Occup. Health Psychol..

[13] Xanthopoulou D., Bakker A.B., Demerouti E., Schaufeli W.B. (2007). The Role of Personal Resources in the Job Demands-Resources Model. Int. J. Stress Manag..

[14] Abualruz H., Rayan A., Al-ghabeesh S., Fawaz M., Jaafeer R., Qutami B., Alyami H. (2024). The Role of Psychological Factors on Improving Work Engagement among Nurses. Front. Psychol..

[15] Zhu L.-L., Wang H.-J., Xu Y.-F., Ma S.-T., Luo Y.-Y. (2023). The Effect of Work Engagement and Perceived Organizational Support on Turnover Intention among Nurses: A Meta-Analysis Based on the Price–Mueller Model. J. Nurs. Manag..

[16] Poku C.A., Bayuo J., Agyare V.A., Sarkodie N.K., Bam V. (2025). Work Engagement, Resilience and Turnover Intentions among Nurses: A Mediation Analysis. BMC Health Serv. Res..

[17] Alilyyani B. (2026). Association between Emotional Intelligence and Turnover Intention among Nurses in Saudi Arabia: A Cross-Sectional Mediation Analysis. BMC Nurs..

[18] Altalhi K., Alilyyani B. (2025). Relationship Between Emotional Intelligence, Turnover Intention, and Resilience Among Emergency Nurses in Saudi Arabia: A Cross-Sectional Study. Nurs. Health Sci..

[19] Catrin C., Wisnu C., Tan P.H.P. (2026). Emotional Intelligence, Work Engagement, and Turnover Intention Among Hospital Nurses: A Hierarchical Component Analysis. J. Keperawatan Indones..

[20] Faul F., Erdfelder E., Buchner A., Lang A.-G. (2009). Statistical Power Analyses Using G*Power 3.1: Tests for Correlation and Regression Analyses. Behav. Res. Methods.

[21] Pérez-Fuentes M.D.C., Molero Jurado M.D.M., Gázquez Linares J.J., Oropesa Ruiz N.F. (2018). The Role of Emotional Intelligence in Engagement in Nurses. Int. J. Environ. Res. Public Health.

[22] Aqtam I., Ayed A., Batran A., Ejheisheh M.A., Melhem R.H., Shouli M. (2025). Work Engagement and Its Association with Emotional Intelligence and Demographic Characteristics among Nurses in Palestinian Neonatal Intensive Care Units. PLoS ONE.

[23] McCloskey J.C., McCain B.E. (1987). Satisfaction, Commitment and Professionalism of Newly Employed Nurses. Image J. Nurs. Scholarsh..

[24] McCloskey J.C. (1990). Two Requirements for Job Contentment: Autonomy and Social Integration. Image J. Nurs. Scholarsh..

[25] Abualrub R.F., Alghamdi M.G. (2012). The Impact of Leadership Styles on Nurses’ Satisfaction and Intention to Stay among Saudi Nurses: Leadership Styles, Satisfaction, and Intention to Stay. J. Nurs. Manag..

[26] Zhu Y., Liu C., Guo B., Zhao L., Lou F. (2015). The Impact of Emotional Intelligence on Work Engagement of Registered Nurses: The Mediating Role of Organisational Justice. J. Clin. Nurs..

[27] Han L., Li R., Wang Y.-M., Liu Y., Bai X. (2025). The Mediating Role of Emotional Intelligence in the Organisational Commitment and Turnover Intention of Clinical Nurses: A Cross-Sectional Study. Nurs. Open.

[28] Xu G., Zeng X., Wu X. (2023). Global Prevalence of Turnover Intention among Intensive Care Nurses: A Meta-analysis. Nurs. Crit. Care.

[29] Ren H., Xue Y., Li P., Yin X., Xin W., Li H. (2024). Prevalence of Turnover Intention among Emergency Nurses Worldwide: A Meta-Analysis. BMC Nurs..

